# *Lactobacillus rhamnosus* GG Activation of Dendritic Cells and Neutrophils Depends on the Dose and Time of Exposure

**DOI:** 10.1155/2016/7402760

**Published:** 2016-07-20

**Authors:** Shirong Cai, Matheswaran Kandasamy, Juwita N. Rahmat, Sin Mun Tham, Boon Huat Bay, Yuan Kun Lee, Ratha Mahendran

**Affiliations:** ^1^Department of Surgery, Yong Loo Lin School of Medicine, National University of Singapore, Singapore 119228; ^2^Department of Anatomy, Yong Loo Lin School of Medicine, National University of Singapore, Singapore 117594; ^3^Department of Microbiology and Immunology, Yong Loo Lin School of Medicine, National University of Singapore, Singapore 117545

## Abstract

This study evaluates the ability of* Lactobacillus rhamnosus* GG (LGG) to activate DC and neutrophils and modulate T cell activation and the impact of bacterial dose on these responses. Murine bone marrow derived DC or neutrophils were stimulated with LGG at ratios of 5 : 1, 10 : 1, and 100 : 1 (LGG : cells) and DC maturation (CD40, CD80, CD86, CD83, and MHC class II) and cytokine production (IL-10, TNF-*α*, and IL-12p70) were examined after 2 h and 18 h coculture and compared to the ability of BCG (the present immunotherapeutic agent for bladder cancer) to stimulate these cells. A 2 h exposure to 100 : 1 (high dose) or an 18 h exposure to 5 : 1 or 10 : 1 (low dose), LGG : cells, induced the highest production of IL-12 and upregulation of CD40, CD80, CD86, and MHC II on DC. In DCs stimulated with LGG activated neutrophils IL-12 production decreased with increasing dose. LGG induced 10-fold greater IL-12 production than BCG. T cell IFN*γ* and IL-2 production was significantly greater when stimulated with DC activated with low dose LGG. In conclusion, DC or DC activated with neutrophils exposed to low dose LGG induced greater Th1 polarization in T cells and this could potentially exert stronger antitumor effects. Thus the dose of LGG used for immunotherapy could determine treatment efficacy.

## 1. Introduction

*Lactobacillus rhamnosus* GG (LGG) is a gram positive lactic acid bacterium that is part of the commensal microflora in humans. It is generally regarded as safe and has been used extensively in food products and health supplements. LGG has been reported to alleviate allergies and dermatitis [1, 2]. Meta-analysis of probiotic supplementation during pregnancy and early infancy indicates a reduced risk ratio of developing eczema in early infancy [3]. A meta-analysis of LGG supplementation showed increased treatment responders in subjects with abdominal pain related gastrointestinal disorders and Irritable Bowel Syndrome [4]. Ohashi et al. also found that long-term consumption of* Lactobacillus casei* was associated with the reduced risk of bladder cancer [5]. LGG was also shown to possess antitumor effects in animal models of bladder cancer [6, 7]. The antitumor effects were comparable to that induced by* Mycobacterium bovis*, bacillus Calmette-Guérin [7]. Intravesical instillations of LGG resulted in an influx of dendritic cells (DCs) and neutrophils [7]. Despite BCG's efficacy it is associated with significant side-effects and less toxic therapies are needed [8]; thus this study further evaluates the immunotherapeutic potential of LGG.

DCs are antigen presenting cells that play an important role in cancer immunotherapy by stimulating cytotoxic T lymphocytes (CTL) and polarizing T helper cells towards a Th1 profile. DC maturation causes enhanced expression of surface costimulatory molecules and production of cytokines and chemokines. However, extensive stimulation of DC can result in DC exhaustion that is characterized by diminished production of IL-12 [9] which is necessary for CTL induction and interferon gamma (IFN*γ*) [10] production. DC exhaustion can be the result of prolonged exposure to a stimulus or exposure to a very high dose of a stimulus either of which scenarios are possible when analyzing the interaction of microbes with immune cells.

Miyazaki et al. [11] showed that, upon inflammation, neutrophils migrate from the site of infection to neighboring lymph nodes where they undergo apoptosis and are taken up by DCs, thus ensuring that neutrophil derived antigens are presented to T cells. Neutrophils are also able to directly transfer antigens to DCs as was demonstrated by Morel et al. [12], studying BCG infected neutrophils.

This work evaluates the impact of the dose of LGG and time of exposure on DC activation in the absence and presence of neutrophils and the consequent stimulation of T cells. The mouse orthotopic tumor model used to assess the intravesical instillation of LGG into the bladder followed the clinical protocol of BCG immunotherapy and was performed over a 2-hour time frame [7]. Thus, this was the minimum time of interaction that was analyzed and 18 h was chosen as the maximum time of interaction as, beyond this time frame, DC viability was greatly reduced after exposure to a high dose of LGG. The death induced by LGG on longer exposure may be a consequence of lactic acid production as observed with cancer cells exposed to LGG [13].

## 2. Materials and Methods

### 2.1. Bacterial Preparation

*L. rhamnosus* GG (National Collections of Industrial and Marine Bacterial Ltd., UK) was streaked onto deMan Rogosa Sharpe (MRS) agar (Difco Laboratories, USA) and incubated at 37°C in 5% CO_2_ [14]. Single colonies were used to produce seed cultures (9 h) which were used to start 50 mL cultures. Bacteria were harvested at the late log phase by centrifugation (1699 ×g for 10 minutes at room temperature) and washed twice with sterile saline (0.85% NaCl). The colony forming units (CFU) were determined by plating serial dilutions of the bacterial samples on MRS agar plates which were incubated at 37°C in 5% CO_2_. BCG Connaught was prepared in the lab as previously described [15].

### 2.2. Preparation of Bone Marrow Derived Neutrophils, DC, and T Cells

Protocols were approved by the Institutional Animal Care and Use Committee (IACUC) at the National University of Singapore. Bone marrow derived neutrophils and DC were generated as previously described [16]. In brief, neutrophils were derived by positive selection with anti-Ly6G microbead kit (Miltenyi Biotec, Germany) and were at least 95% positive for Ly6G, by flow cytometry. DCs were obtained from the bone marrow derived cells after 9 days of culture (with fresh media replacement every other day) in RPMI 1640 supplemented with 10% heat-inactivated FCS, 50 *μ*M 2-mercaptoethanol, 1% penicillin, streptomycin, glutamine, MEM (minimum essential medium), and 0.1% sodium pyruvate with 40 ng/mL of GM-CSF (BD Bioscience, USA). The DCs were at least 95% positive for CD11c, by flow cytometry. The media used for both neutrophil and DC experiments were DMEM supplemented with 10% fetal bovine serum (Hyclone, USA), 2 mM L-glutamine (Gibco, Japan), 50 *μ*g/mL penicillin G (Sigma Aldrich, USA), and 50 *μ*M of 2-mercaptoethanol (Merck, Germany) with the addition of 20 ng/mL of GM-CSF for the culture of dendritic cells.

T lymphocytes were isolated from spleens of naive C57BL/6 mice and enriched with the EasySepTM T cell isolation kit (STEMCELL Technologies, Vancouver, Canada). The desired fraction was about 95–98% CD3 positive.

### 2.3. Neutrophil, Dendritic Cell LGG Coculture, and Blocking of IL-10 and COX-2

The LGG to cell ratios of 5 : 1 and 10 : 1 were defined as exposure to low dose LGG, while exposure to a ratio of 100 : 1 was defined as exposure to high dose LGG. Neutrophils (5 × 10^5^) and DCs (2.5 × 10^5^) were cocultured with LGG at bacteria to mammalian cell ratios of 5 : 1, 10 : 1, and 100 : 1 for 2 h in 24-well plates, before 200 *μ*g/mL of gentamicin was added for 2 h at 37°C to kill extracellular LGG. Cells were washed thrice with PBS to remove extracellular bacteria and then neutrophils were incubated with DCs (2.5 × 10^5^) for 18 h and DCs were incubated in fresh media for 18 h. Untreated neutrophils and DCs were evaluated as controls. All controls were given the same treatment as above. For 2.5 × 10^5^ DCs the bacteria CFU that corresponded to 5 : 1, 10 : 1, and 100 : 1 were 1.25 × 10^6^, 2.5 × 10^6^, and 25 × 10^6^ CFU. The neutrophils (5 × 10^5^ cells) were treated with 2.5 × 10^6^, 5 × 10^6^, and 50 × 10^6^ CFU of LGG that corresponded to 5 : 1, 10 : 1, and 100 : 1, LGG to cells. For the DC 18 h experiment the DCs were exposed to LGG for 18 h. Similarly cells were treated with BCG at a 5 : 1 ratio. The supernatants were assayed for TNF-*α*, IL-12p70, and IL-10 (eBioscience, San Diego, USA) and prostaglandin E2 (PGE2) (Cayman Chemical, USA) by ELISA using a GENios Pro*™* microplate reader (Tecan, Switzerland). The cells were harvested in PBA (PBS with 1% bovine serum albumin and 0.01% sodium azide) for flow analysis of surface markers.

IL-10 and COX-2 were inhibited by pretreatment with 400 ng/mL of anti-IL-10 antibody (Biolegend, San Diego, USA) and 10 *μ*M of NS398 (Sapphire Bioscience, Australia), respectively, for 30 mins at 37°C prior to addition of bacteria and then further incubated for another 18 hours. The respective isotype and solvent controls were included for comparison. The efficacy of the blocking was confirmed by ELISA.

### 2.4. Flow Cytometry and Antibodies

Fixed DCs were double stained with anti-CD11c antibody and antibodies of the following surface markers: CD40, CD80, CD83, CD86, and MHC II (Biolegend) or the respective isotype controls in PBA (1x PBS with 1% BSA, 0.05% sodium azide) for 20 mins at 4°C. After that the cells were washed once and resuspended with PBA before they were analyzed with BD FACS Canto using FACS Diva software (Becton Dickinson, USA).

### 2.5. DC-T Cell Coculture

DCs or T cells were resuspended in LDA medium (NCTC 109 and RPMI 1640 [1 : 1]), supplemented with 10% heat-inactivated FCS, 10 mM L-glutamine, 1 mM oxaloacetic acid, 0.2 U of bovine insulin per mL, and 50 *μ*M 2-mercaptoethanol. Naive T cells (1.0 × 10^7^ cells/mL) were cultured with untreated DCs (1.0 × l0^5^ cells/mL) or DCs stimulated with lactobacilli for 2 h and 18 h (treated as described above), in 200 *μ*L of LDA medium in 96-well U-bottom plates at 37°C under 5% CO_2_ for 5 days. After 5 days, supernatants were harvested and analyzed for IFN gamma or IL-2.

### 2.6. Statistical Analysis

One-way ANOVA with post hoc Bonferroni test was used to analyze all the data except for the comparison of cytokine profile of the treatment groups with anti-IL-10 antibody or NS398 and their respective controls, which were analyzed with Student's *t*-test. A significant difference was taken to exist when the *p* value was <0.05.

## 3. Results

### 3.1. LGG Dose, Exposure Time, and Neutrophils Modulate DC and Neutrophil Maturation and Viability

A short exposure (2 h) to low dose LGG (LGG to cell ratios of 10 : 1 and 5 : 1) reduced DC viability slightly (91.7 ± 2.0% and 94.7 ± 1.7%, resp.) and there was little loss in viability even at 18 h. Exposure to activated neutrophils had a similar effect. However at a prolonged exposure of 18 h to high dose LGG (100 : 1 LGG to DC ratio), there was reduced DC viability (63.7 ± 1.8%).

About 50% of neutrophils were dead (apoptotic and necrotic death) at 18 h after the initial 2 h exposure to LGG regardless of dose. But in contrast there was increased LGG internalized with exposure to increased LGG dose. At a 5 : 1 ratio of LGG : neutrophils there were 228 ± 51 CFU internalized/5 × 10^5^ neutrophils and this almost doubled after exposure to 10 : 1 and more than doubled again after exposure to 100 : 1 LGG to neutrophils. The LGG in the neutrophils were still viable at 18 h after internalization.

Activation markers on naïve DC were examined and after exposure to LGG there was a significant increase in all markers with respect to naïve DC, Table 1. As a further control DCs were also exposed to BCG, Table 1. After high dose LGG exposure for 2 h, there was significantly (*p* < 0.05) higher expression of CD80, CD86, and CD40 compared to low dose LGG. But at 18 h coincubation with LGG, expression of CD86 and CD40 was significantly reduced (*p* < 0.05) after exposure to high dose LGG compared to low dose LGG. DCs cocultured with neutrophils, activated with low doses of LGG for 2 h, showed higher expression of CD86 and reduced CD83 compared to DCs exposed directly to low dose LGG for 2 h. DC exposed to BCG at a dose of 5 : 1, for 2 h or 18 h, or stimulated by neutrophils activated with BCG did not show a difference in surface marker expression. In contrast LGG at the same dose showed changes in the expression of CD83 and CD86.

Neutrophils cultured with LGG showed decreased MHC class I expression, no increase in MHC class II expression, and an increase in CD11b expression when placed directly in contact with LGG. CD11b is an activation marker for neutrophils and has been shown to activate DC maturation via interaction with DC-SIGN [17].

MHC class II mean fluorescence index (MFI) showed a doubling on exposure to low dose LGG or activated neutrophils, Table 2. The MFI for MHC class II, CD40, and CD80 was decreased after exposure to DC activated for 18 h, with either high dose LGG or neutrophils activated with high dose LGG (*p* < 0.05). But the reverse was true for CD83 when DCs were exposed to high dose LGG for 18 h (*p* < 0.05), Table 2. The MFI of CD86 did not vary with treatment conditions.

### 3.2. LGG Dose, Exposure Time, and Neutrophils Modulate DC Cytokine Production

**Figure 1:**
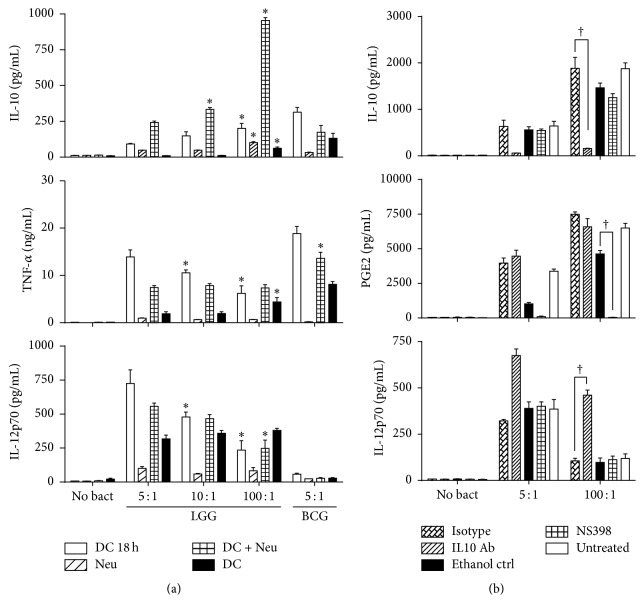
LGG and BCG induced dose and time dependent effects on DC cytokine production by direct or indirect stimulation via neutrophils. LGG was assessed at 5 : 1, 10 : 1, and 100 : 1 ratios and BCG at 5 : 1 ratios. (a) Production of IL-10, TNF-*α*, and IL-12p70 after 18 h of continuous coculture of DCs with LGG/BCG (white bar), 2 h of exposure of DCs (black bar) or neutrophil (striped bar) to LGG/BCG, followed by 18 h of bacteria free incubation and 18 h of DC coculture with neutrophils pretreated with LGG/BGC for 2 h (crisscross bar). “*∗*” indicates a significant difference (*p* < 0.05) compared to low dose (5 : 1). For BCG “*∗*” indicates a significant difference from neutrophils and for TNF-*α* significance with respect to DC. Data are presented as the mean ± SEM. (b) Neutrophils were prestimulated with low (5 : 1) and high (100 : 1) dose of LGG for 2 h before they were cocultured with DC for 18 h in the presence and absence of a COX-2 inhibitor, NS398 (crisscross bar); COX-2 inhibitor solvent control (black bar); IL-10 neutralizing antibody (IL-10 Ab) (striped bar); and the isotype control for the antibody (double striped bar). The impact on PGE_2_, IL-10, and IL-12p70 secretion is shown. “†” indicates a significant difference from the respective controls (*p* < 0.05). Data are presented as the mean ± SEM.

More IL-10 was produced after exposure to high dose LGG (Figure 1(a)) for 2 h and 18 h and via neutrophil mediated activation. TNF-*α* production was higher in DC exposed to high dose LGG (Figure 1(a)) for 2 h, but at 18 h, DC exposed to low dose LGG produced more TNF-*α*. Both indirect (via neutrophils) and direct DC activation for 18 h resulted in more IL-12p70 production after low dose LGG exposure (Figure 1(a)). With a short 2 h exposure to LGG, IL-12p70 production was independent of the bacterial dose.

Neutrophils stimulated with LGG produced IL-12p70 and TNF-*α* and very little IL-10. LGG activated neutrophils (2 h) induced more IL-10, TNF-*α*, and IL-12p70 production in DC compared to DC exposed directly to low dose LGG for 2 h. At high dose LGG there was no significant difference in IL-12p70 and TNF-*α* production, whether the DC was stimulated directly with LGG or with activated neutrophils.

In contrast, when DC and neutrophils were exposed to BCG at a 5 : 1 ratio [12], there was comparable production of IL-10 from all groups except DC exposed to BCG for 18 h. The amount of IL-12p70 produced after BCG stimulation was at least 10-fold lower than that produced by LGG. The TNF-*α* response was comparable to LGG (Figure 1(a)).

### 3.3. IL-12p70 Production after Exposure to LGG Stimulated Neutrophils Is Dependent on IL-10

Since, at high dose LGG, IL-10 production is significantly higher in DCs, as well as DC treated with activated neutrophils, we determined if the dose dependent effects on IL-12 production were due to IL-10 levels. PGE_2_ is known to modulate IL-10 expression; induce indoleamine 2,3-dioxygenase (a potent suppressor of DC function); and modulate chemokine production and DC maturation and IL-12p70 production [18–20]. Therefore PGE_2_ and IL-10 production/function were inhibited individually and the impact on IL-12p70 production was monitored, Figure 1(b). There was a significant increase in PGE_2_ levels on DC stimulation with high rather than low dose LGG. At the concentration of NS398 that completely blocked the production of PGE_2_ (Figure 1(b)) there was no significant effect on either IL-10 or IL-12p70 levels, Figure 1(b). Blocking IL-10 with a neutralizing antibody caused a sharp increase in IL-12p70 production, Figure 1(b). This corresponded to 2.1- and 4.4-fold increases, respectively, in DC stimulated with neutrophils activated with low and high dose LGG. Expression of surface markers on dendritic cells was not significantly affected by the presence of either the anti-IL-10 antibody or NS398 (data not shown).

### 3.4. T Cell Activation Is Dependent on LGG to DC Ratios and Time of Exposure

**Figure 2:**
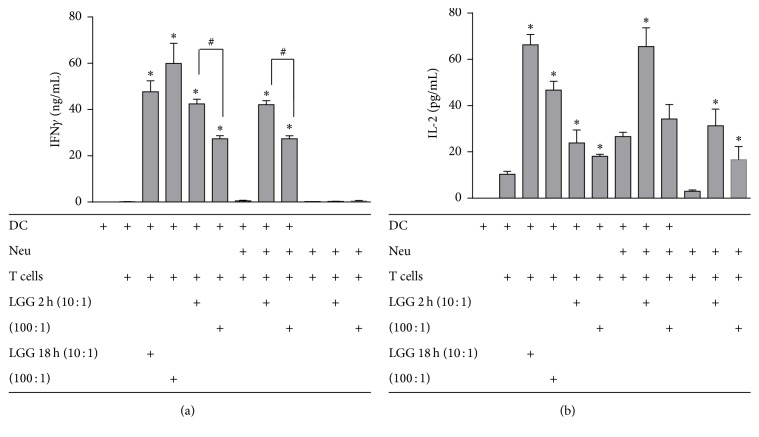
T cell activation is dependent on the dose of LGG used to stimulate DC or neutrophils. IFN*γ* (a) and IL-2 (b) production by T cells after 5 days of coculture with DC, neutrophil, or DC-neutrophil (stimulated with low or high dose of LGG for 2 h and then DC for 18 h). Data are presented as the mean ± SEM. “*∗*” indicates a significant difference compared to their respective no LGG controls (*p* < 0.05). “#” indicates significant difference between high and low dose of LGG treatment (*p* < 0.05).

Neutrophils stimulated with LGG did not induce IFN*γ* production by T cells (Figure 2(a)) but induced IL-2 production (Figure 2(b)). DCs stimulated with LGG activated neutrophils induced a significant increase (*p* < 0.05) in IFN*γ* production (Figure 2(a)) and a slight increase in IL-2 production by T cells (Figure 2(b)) similar to DC directly activated with low dose LGG. The DC-neutrophil-T cell triple cell culture by itself induced IL-2 production. A dose dependent effect was clearly seen with IFN*γ* production (*p* < 0.05) (Figure 2(a)) which was consistent with the decrease in IL-12p70 production that was observed earlier. Direct or indirect DC activation with low dose LGG for 2 h induced more IFN*γ* and IL-2 production by T cells (*p* < 0.05 for IFN*γ*) compared to high dose LGG, Figures 2(a) and 2(b). At 18 h the differential effect of the dose was lost for IFN*γ*.

## 4. Discussion

On activation by LGG, there was an increase in the percentage of DC expressing CD40, CD80, and CD86 with increasing dose. But only CD40 had a significant increase in MFI (*p* < 0.05), indicating increased protein expression. CD86 and CD80 interact with CD28 on T cells while CD40 binds to the CD40 Ligand on T cells to induce T cell activation. On prolonged exposure (18 h) to high dose LGG, the percentage of cells expressing CD86 and CD40 and the MFI of CD40, CD86, and CD80 were reduced. Thus, these DCs may not be as able to activate T cells as efficiently; that is, there is a point beyond which LGG dose can be inhibitory to DC activation. A similar effect was observed with* L. casei *[21] which also has antitumor effects [22] and other commensal lactobacilli such as* L. gasseri, L. johnsonii,* and* L. reuteri *[23].

Different* Lactobacillus* species induce variable levels of IL-10, IL-12, and TNF-*α* via Toll-like receptor (TLR) dependent activation of DC [24]. Indirect DC stimulation via LGG activated neutrophils showed no difference in TNF-*α* production with increasing dose. But primary interaction between DC and LGG showed dose dependent effects. This could be due to TLR engagement and phagocytosis [25]. LGG is known to adhere to epithelial cells with greater ability than other* Lactobacillus* species. Such binding to DC may also increase the cellular signals triggered by direct interaction with DC. Tytgat et al. found that LGG pili S SpaCBA could interact with DC-SIGN and that blocking this interaction reduced DC cytokine production [26]. DC-SIGN also modulates TLR activation and it is possible that LGG pili interaction with DC-SIGN could have modulated TLR activated cytokine production. An 18 h exposure to low dose LGG produced more TNF-*α* and IL-12 than exposure to high dose LGG. It is likely that prolonged exposure led to increasing phagocytosis of LGG with time which resulted in triggering the downregulation of TNF-*α* and IL-12 production. IL-12 production is TLR2 independent [25] but TLR9 [27], MyD88, and ROS [28] dependent. Phagocytosis of LGG is important, as is the presence of undigested bacteria [29]. However, with a short exposure to LGG there was a dose dependent effect only on TNF-*α* production which might reflect TLR2 engagement [25].

As neutrophils are generally the first to encounter microbes and move to the lymph nodes to educate DC [30], we evaluated the dose effect of LGG on the ability of neutrophils to activate DC. Stimulating DC with LGG treated neutrophils exposed to low dose LGG induced higher CD86 than direct stimulation of DCs with LGG. Neutrophils cultured with high dose LGG induced a decrease in the MFI of CD40, CD80, and CD83 (*p* < 0.05) on DCs. Though the number of neutrophils undergoing apoptosis was similar at all doses of LGG the number of internalized LGG increased with dose of LGG. The latter may have resulted in increased LGG or LGG components being transferred to DC [11, 12] causing strong stimulation of DC and consequently DC exhaustion. In line with this hypothesis, IL-12p70 and TNF-*α* production were much higher when the DCs were cultured with neutrophils activated with low dose LGG. DCs are known to internalize apoptotic cells [31] which like necrotic cells are able to stimulate DC [32]. Phagocytosis of apoptotic cells induces anti-inflammatory signals [33] such as the high levels of IL-10 which was found in this study.

IFN*γ* production was higher in T cells cocultured with DC and neutrophils treated with low dose LGG for 2 h rather than high dose LGG. Similarly, when DCs were treated with LGG at 200 : 1 bacteria to cell ratio, phenotypic maturation and cytokine production but not Th1 polarization were observed [21]. Instead, the CD4^+^ cells were converted to hyporesponsive T cells that secrete low IFN*γ*. Thus, for optimal T cell activation, low dose LGG is overall the better therapeutic option.

Prolonged stimulation of DCs (for 24 h or longer) can result in the loss of the ability to produce cytokines like IL-12, which is termed DC exhaustion [9, 34]. These “exhausted” DCs tend to induce Th2 cell differentiation. Langenkamp et al. reported that the optimal temporal window to induce DC maturation in order to have sustained IL-12 production for cancer immunotherapy is narrow, with a time frame of 10–18 h [9], but our results indicate that a 2 h exposure is sufficient for DC maturation. Further LGG was much better at inducing IL-12p70 production than BCG, the current standard therapy for bladder cancer.

IL-10 is widely reported to downregulate DC maturation [35, 36] and its ability to activate T cells [37] as well as induce DC apoptosis [38]. PGE_2_, a potent inducer of IL-10 [20], was also found to be produced in greater amounts when DCs were stimulated with neutrophils treated with high dose LGG. Neutralization of IL-10 substantially increased the IL-12p70 production. However, it was still lower than the levels produced by DC coculture with neutrophil stimulated with low dose LGG, suggesting that there are other inhibitory factors aside from IL-10.

## 5. Conclusion

Low dose LGG stimulates DC to induce greater Th1 polarization in T cells compared to high dose LGG. Thus, low dose LGG would potentially be able to exert stronger antitumor effects. In mice LGG (1 × 10^8^ CFU/100 *μ*L) was effective at reducing tumor growth with comparable efficacy to BCG Connaught (1 × 10^7^ CFU/mL) [7]. The former is roughly in the range of a 100 : 1, LGG to cells for 2 h. Thus future analysis should consider the effect of a 10-fold lower dose of LGG as an immunotherapeutic agent. The dose response is an important consideration if LGG is to be used for human bladder cancer therapy.

## Figures and Tables

**Table 1:** Surface marker expression on DC after direct and indirect exposure via neutrophils to BCG and LGG.

Marker	DC	Percentage of CD11c^+^ cells				
		Control	5 : 1 (BCG)	5 : 1 (LGG)	10 : 1 (LGG)	100 : 1 (LGG)
CD40	2 h	6.0 ± 3.3	22.5 ± 3.3	32.1 ± 4.9	32.7 ± 0.3	50.7 ± 2.6^b^
	18 h	2.2 ± 0.5	19.3 ± 5.4	20.5 ± 6.9	19.0 ± 6.8	7.0 ± 0.8^b^
	+neutrophils (2 h)	2.2 ± 0.4	27.8 ± 3.5	22.9 ± 5.2	25.4 ± 8.7	10.4 ± 2.0^b^

CD80	2 h	9.9 ± 1.4	39.3 ± 10.4	18.2 ± 0.8	18.1 ± 3.3	34.0 ± 4.2^b^
	18 h	9.6 ± 1.6	30.7 ± 7.5	17.9 ± 0.5	19.7 ± 2.5	19.6 ± 0.8
	+neutrophils (2 h)	6.1 ± 1.6	33.8 ± 8.9	21.2 ± 5.0	16.6 ± 7.6	16.1 ± 7.8

CD83	2 h	6.5 ± 0.2	29.6 ± 5.7	38.4 ± 3.7	32.7 ± 1.3	36.2 ± 4.7
	18 h	5.1 ± 1.5	27.5 ± 10.5	8.4 ± 1.9	4.8 ± 0.8	6.5 ± 1.4
	+neutrophils (2 h)	3.6 ± 0.2	31.2 ± 4.8	9.8 ± 1.3	8.0 ± 0.8	11.1 ± 2.8

CD86	2 h	17.0 ± 0.3	72.0 ± 5.4	37.1 ± 8.1	41.6 ± 1.4	58.5 ± 6.1^b^
	18 h	14.8 ± 1.9	71.0 ± 7.9	57.6 ± 0.1	56.5 ± 1.3	29.7 ± 6.0^b^
	+neutrophils (2 h)	13.7 ± 0.2	68.0 ± 4.8	68.3 ± 6.4	76.5 ± 4.4	54.9 ± 3.8^b^

MHC class II	2 h LGG	31.7 ± 1.8	61.9 ± 2.5	69.9 ± 8.8	70.7 ± 12.8	77.8 ± 11.1
	18 h LGG	32.6 ± 2.7	59.2 ± 6.1	58.4 ± 12.5	52.9 ± 7.2	57.4 ± 11.6
	+neutrophils (2 h)	34.7 ± 10.1	55.3 ± 10.2	58.5 ± 12.9	52.1 ± 6.3	64.0 ± 9.4

^b^ *p* < 0.05 compared to groups treated with 5 : 1 and 10 : 1 LGG, respectively.

Data are presented as the mean ± SEM.

**Table 2:** LGG dose and time of exposure modulated the MFI of DC surface markers.

Marker	Group	Control	5 : 1	10 : 1	100 : 1
MHC class II	DC 2 h	1191 ± 10	2522 ± 2^a^	2545 ± 94^a^	2978 ± 245^a^
	DC 18 h	1303 ± 37	2066 ± 154^a^	2124 ± 29^a^	1530 ± 3^bc^
	DC + neutrophils	1191 ± 28	2010 ± 107^a^	2352 ± 78^a^	1946 ± 80^ac^

CD40	DC 2 h	539 ± 1	629 ± 1^a^	654 ± 1^a^	684 ± 6^abc^
	DC 18 h	489 ± 42	735 ± 27^a^	721 ± 1^a^	370 ± 8^bc^
	DC + neutrophils	437 ± 61	576 ± 15	642 ± 18^a^	390 ± 21^c^

CD83	DC 2 h	465 ± 20	417 ± 11	554 ± 79	452 ± 22
	DC 18 h	668 ± 20	506 ± 33^a^	681 ± 7^b^	702 ± 12^b^
	DC + neutrophils	704 ± 34	609 ± 61^a^	441 ± 54^a^	351 ± 3^ab^

CD80	DC 2 h	515 ± 4	537 ± 5	516 ± 14	568 ± 15^ac^
	DC 18 h	513 ± 3	544 ± 12	535 ± 10	478 ± 28^b^
	DC + neutrophils	476 ± 6	613 ± 16^a^	553 ± 10^a^	478 ± 21^bc^

^a^ *p* < 0.05 compared to control with no bacteria.

^b, c^ *p* < 0.05 compared to groups treated with 5 : 1 and 10 : 1 LGG, respectively.

Data are presented as the mean ± SEM.

## Abbreviations

LGG: * Lactobacillus rhamnosus* GG
BCG: Bacillus Calmette-Guérin
DCs: Dendritic cells.
